# Antibacterial activity of silver nanoparticles obtained by pulsed laser ablation in pure water and in chloride solution

**DOI:** 10.3762/bjnano.7.40

**Published:** 2016-03-18

**Authors:** Brunella Perito, Emilia Giorgetti, Paolo Marsili, Maurizio Muniz-Miranda

**Affiliations:** 1 Department of Biology, University of Florence, Via Madonna del Piano 6, Sesto Fiorentino (FI) 50019, Italy; 2 Institute of Complex Systems (ISC) CNR, Via Madonna del Piano 10, Sesto Fiorentino (FI) 50019, Italy; 3 Department of Physics “Enrico Fermi”, University of Pisa, Largo Bruno Pontecorvo 3, Pisa, 56127, Italy,; 4 Department of Chemistry “Ugo Schiff”, University of Florence, Via della Lastruccia 3, Sesto Fiorentino (FI) 50019, Italy

**Keywords:** antibacterial activity, colloid, laser ablation, nanoparticles, silver

## Abstract

Silver nanoparticles (AgNPs) have increasingly gained importance as antibacterial agents with applications in several fields due to their strong, broad-range antimicrobial properties. AgNP synthesis by pulsed laser ablation in liquid (PLAL) permits the preparation of stable Ag colloids in pure solvents without capping or stabilizing agents, producing AgNPs more suitable for biomedical applications than those prepared with common, wet chemical preparation techniques. To date, only a few investigations into the antimicrobial effect of AgNPs produced by PLAL have been performed. These have mainly been performed by ablation in water with nanosecond pulse widths. We previously observed a strong surface-enhanced Raman scattering (SERS) signal from such AgNPs by “activating” the NP surface by the addition of a small quantity of LiCl to the colloid. Such surface effects could also influence the antimicrobial activity of the NPs. Their activity, on the other hand, could also be affected by other parameters linked to the ablation conditions, such as the pulse width. The antibacterial activity of AgNPs was evaluated for NPs obtained either by nanosecond (ns) or picosecond (ps) PLAL using a 1064 nm ablation wavelength, in pure water or in LiCl aqueous solution, with *Escherichia coli* and *Bacillus subtilis* as references for Gram-negative and Gram-positive bacteria, respectively. In all cases, AgNPs with an average diameter less than 10 nm were obtained, which has been shown in previous works to be the most effective size for bactericidal activity. The measured zeta-potential values were very negative, indicating excellent long-term colloidal stability. Antibacterial activity was observed against both microorganisms for the four AgNP formulations, but the ps-ablated nanoparticles were shown to more effectively inhibit the growth of both microorganisms. Moreover, LiCl modified AgNPs were the most effective, showing minimum inhibitory concentration (MIC) values in a restricted range of 1.0–3.7 µg/mL. An explanation is proposed for this result based on the increased surface reactivity of the metal surface due to the presence of positively charged active sites.

## Introduction

The interest in nanoscale metal particles is constantly growing as they find wide application in diverse fields ranging from sensing [1–3], medicine [4], catalysis [5–8], to astrobiology [9–10] and many others. In particular, silver nanoparticles (AgNPs) have increasingly gained importance as promising new antimicrobial agents with application in several biomedical fields, in water and air filtration, as well as in conservation of cultural heritage [11–14]. Although the mode of action of AgNPs against microorganisms is not yet fully understood, it is generally believed that different mechanisms determine the antimicrobial activity of AgNPs based on both the release of silver ions and the nanoparticle characteristics [15–16]. Some of these proposed mechanisms include: (a) the direct contact between NPs and the microbial cell, which disturbs the power functions of the cell membrane and causes structural damage; (b) the generation of reactive oxygen species (ROS), which damage the cell membrane; and (c) the interference with DNA replication and inhibition of enzymes and other proteins [13,17–20]. These multiple, synergic mechanisms of cytotoxic activity reduce the likelihood that the microorganisms develop resistance against the silver compounds [21]. Consequently, AgNPs are very attractive as antimicrobials, due to the worldwide crisis of bacterial resistance to conventional, narrow-target antibiotics [19].

AgNPs have been synthesized by following various physical, chemical and biological pathways [22–23]. Their microscopic, physical and chemical properties have been found to be closely related to the experimental preparation procedures, the interaction of metal ions with reducing agents, as well as the adsorption of stabilizers [22]. Furthermore, the presence of residual reagents or by-products from these methods can lead to the irreproducibility of desired NP characteristics [24], while their potential toxicity hinders further biological applications [25].

These drawbacks can be overcome by synthesizing the NPs using pulsed laser ablation in liquid (PLAL). In fact, PLAL is a physical approach that permits preparation of stable metal colloids in pure solvents without the use of capping or stabilizing agents [26–27]. The NPs are obtained by focusing a pulsed laser beam onto a metallic target immersed in a liquid, which can be a pure solvent or a solution containing capping and stabilizing molecules, when required. In the first case, the surface of PLAL-synthesized NPs is considered to be “clean” and the colloids will be free from reaction by-products. With this method, it is possible to isolate the effect of Ag on living cells (and, in particular, on bacteria) from that of other compounds.

Most of the studies on the bactericidal effect of AgNPs concern NPs obtained by wet chemical methods. From studies using AgNPs with different sizes, it has been demonstrated that their antibacterial activity decreases with increasing particle size. The effect of 1–100 nm AgNPs on Gram-negative bacteria was studied by Morones et al. with HR-TEM analysis [13]. They concluded that only AgNPs with a diameter <10 nm are able to interact with the bacteria. Additionally, bacterial growth inhibition was shown to be more effective with AgNPs with an average diameter of less than 10 nm. The lowest minimum inhibitory concentration (MIC) values for Gram-positive as well as Gram-negative bacteria were correlated to the smallest nanoparticles used (5 nm and 7 nm) in different studies [28–30].

In contrast, only a few investigations have been performed that analyze the bactericidal properties of AgNPs produced by PLAL. In spite of the superior surface cleanliness and the absence of capping agents, which could induce a potential shielding effect on the antimicrobial activity, the obtained MIC values were notably in the same range as those of the AgNPs prepared by wet chemical methods [31]. As a matter of fact, as very recently observed by some of us [32], AgNPs synthesized by PLAL in pure water are coated by a thin oxide layer that, in the case of Raman experiments, impairs their ability to induce a strong increase of the Raman response of molecular adsorbates in the SERS (Surface Enhanced Raman Scattering) effect. A strong SERS signal from such AgNPs can be obtained by “activating” the NP surface by addition of a small quantity of LiCl to the colloid. In addition, a sizeable catalytic effect has also been observed in chloride-activated Ag colloids [24]. It is therefore reasonable to expect that such surface effects can also influence the antimicrobial activity of the AgNPs. With this in mind, AgNPs with an average diameter less than 10 nm were synthesized either by ns or ps PLAL in pure water or in aqueous solutions of LiCl using a 1064 nm ablation wavelength. Then, the obtained AgNPs were characterized and tested for antimicrobial activity against *Escherichia coli* and *Bacillus subtilis* as references for Gram-negative and Gram-positive bacteria, respectively.

## Results

### AgNP characterization

AgNPs were prepared by PLAL. The experimental setup has been previously described by Giorgetti et al. [31], and the specific fabrication conditions are reported in the Experimental section. The list of samples with their characteristics is shown in Table 1.

**Table 1:** Summary of AgNP colloids and of their characteristics.

Sample	Absorbance at 400 nm (OPL = 1 mm)	Pulse width (s)	Pulse energy (mJ)	Fluence on target (J/cm^2^)	Zeta potential (mV)	Average NP diameter (σ_−_ /σ_+_)^a^ (nm)	Ag concentration (μg/mL)

AgNPsH_2_Ops	0.9	25 × 10^−12^	15	0.7	−33 ± 8 (57%) −53 ± 5 (43%)	2.2 (1.5/4.4)	84.1
AgNPsH_2_Ons	2.3	25 × 10^−9^	100	4	−50 ± 7 (51%) −25 ± 6 (49%)	2.2 (1.3/3.0)	184.4
AgNPsLiClps	0.4	25 × 10^−12^	15	0.7	−55 ± 7 (100%)	3.3 (2.1/6.2)	47.0
AgNPsLiClns	1.5	25 × 10^−9^	100	4	−35 ± 7 (61%) −68 ± 7 (39%)	0.9 (0.5/1.2) 6.1 (3.1/6.4)	74.1

^a^The left (σ_−_) and right (σ_+_) 1/*e* half widths of the size distribution, respectively.

**Figure 1:**
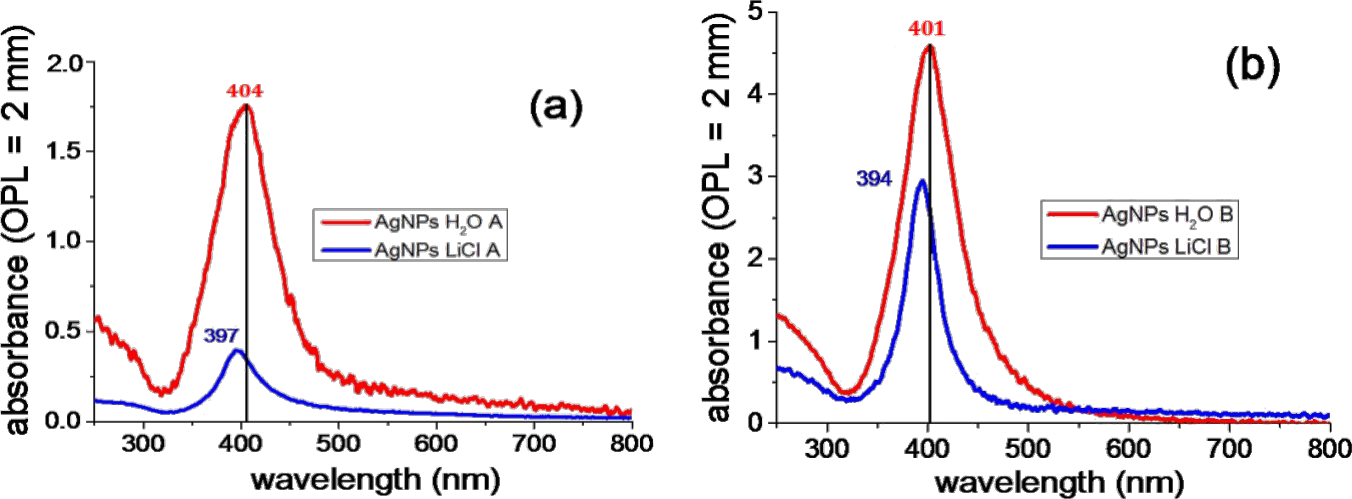
UV–vis absorption spectra of AgNPs in colloidal suspensions obtained with (a) ps and (b) ns laser ablation.

Figure 1 reports the UV–vis absorption spectra of the colloidal AgNPs samples obtained with ps laser ablation (Figure 1a) and with ns (Figure 1b) pulses, namely ps and ns samples. In particular, the figure compares the spectra of the samples obtained in pure water (red line) with those of the samples prepared in 1 mM LiCl aqueous solution (blue line). In the latter case, the plasmon resonance appears blue-shifted in both ps and ns samples. Such a shift could be attributed either to a change in the dielectric constant of the liquid environment or, more reasonably, to the different oxidation grade of the NP surfaces, where the NPs obtained in the presence of LiCl would be less oxidized [32].

In addition, the plasmon bands of the silver nanoparticles obtained by ps ablation in both pure water and LiCl solution appear significantly red-shifted compared to those observed for colloids obtained by ns ablation. This result could also be attributed to a difference in silver oxide content on the AgNPs. It is known that thermal evaporation dominates in the process of ns ablation [33], while a nonthermal mechanism (attributable to photoionization [34]) is predominant in the ps ablation process. Because Ag_2_O dissociates above 550 K, it is reasonable to expect more silver oxide in ps-ablated material. Moreover, the presence of a larger content of silver oxide on the surface of ps-ablated nanoparticles with respect to those ns-ablated was previously ascertained by means of UV–vis absorption experiments and theoretical modelling for Ag colloids obtained by laser ablation [32].

In order to study the long-term colloidal stability and the electrical characteristics of the NP surface, the zeta potential was measured. The obtained values are reported in Table 1. Apart from the AgNPsLiClps sample, all samples exhibited a bimodal distribution of the zeta potential. In all cases, the zeta potential is strongly negative, indicating excellent long-term stability of the NPs, as already assessed by Giorgetti et al. [32]. Furthermore, as expected, the adsorption of chloride ions shifts the zeta potential of the samples obtained in LiCl solution towards more negative values. The negative zeta potential of the colloids obtained in water is due to the adsorption of hydroxide anions from the aqueous medium, whereas for the colloids obtained in LiCl solution it is due to the preferential adsorption of chloride anions on the silver surface [35].

**Figure 2:**
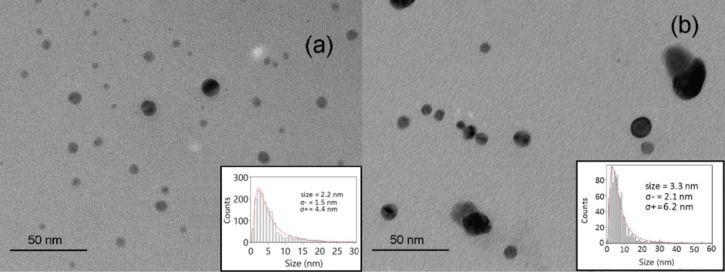
Typical TEM image and size (average diameter) distribution of (a) AgNPsH_2_Ops and (b) AgNPsLiClps.

We have also performed a TEM investigation on the AgNPs obtained in the four different procedures: by ps or ns laser ablation and in pure water or in LiCl solution. The reported TEM images represent the most significant images for the four samples, and the size distribution (reported as inserts in the same figures) were obtained from the diameter evaluation of more than 2000 nanoparticles. Figure 2a shows the morphological characteristics of the sample AgNPsH_2_Ops. Apart from a small amount of large NPs with average diameter around 10 nm, the majority of NPs are small, with a 2.2 nm diameter and σ_−_ = 1.5 nm and σ_+_ = 4.4 nm, which are the left and right 1/*e* half widths of the size distribution, respectively. The addition of LiCl to the ps ablation environment increases the presence of large NPs in the sample. Figure 2b shows a typical TEM micrograph of the sample AgNPsLiClps and the corresponding size distribution, which includes a non-negligible amount of NPs larger than 10 nm. In this case, the statistical analysis of TEM data returned a size distribution with peak diameter of 3.3 nm, σ_−_ = 1 nm and σ_+_ = 3 nm.

**Figure 3:**
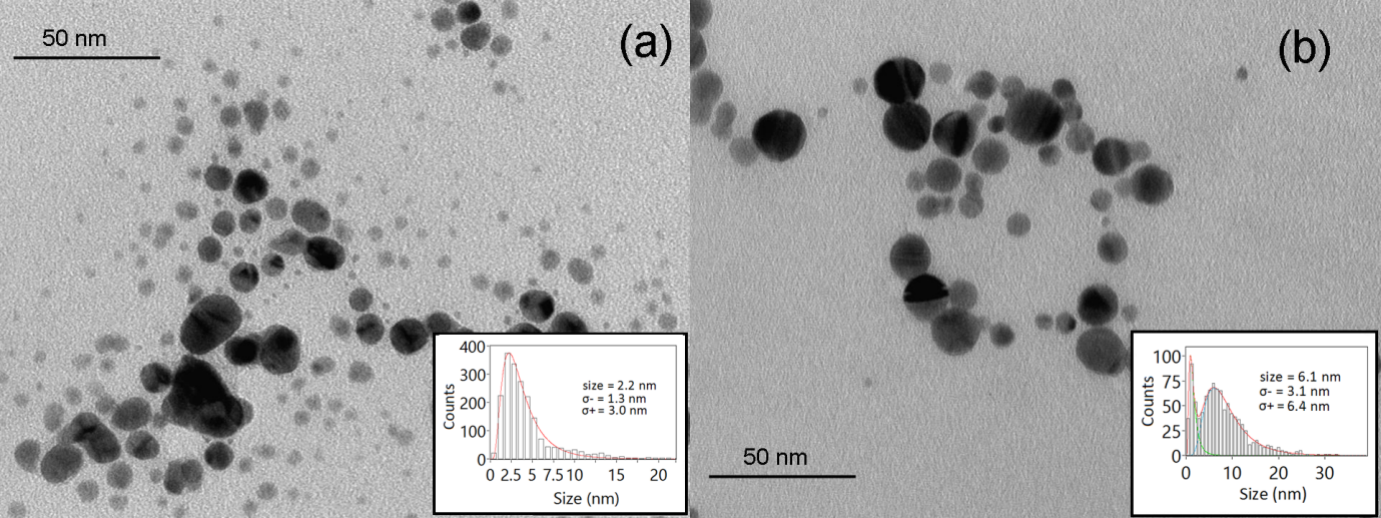
Typical TEM image and size (average diameter) distribution of (a) AgNPsH_2_Ons and (b) AgNPsLiClns.

Analogous to the corresponding ps sample, the AgNPsH_2_Ons sample exhibited a monomodal size distribution, as shown in Figure 3a (diameter 2.2 nm; σ_−_ = 1.3 nm; σ_+_ = 3.0 nm). Also, for samples produced with ns PLAL, the addition of LiCl to the ablation environment caused an increase in NP size, more markedly than for the ps-ablated sample. The ns-ablated sample had a bimodal size distribution, where larger NPs (diameter 6.1 nm; σ_−_ = 3.1 nm; σ_+_ = 6.4 nm) were statistically more prominent than smaller NPs (diameter 0.9 nm; σ_−_ = 0.5 nm; σ_+_ = 1.2 nm), as reported in Figure 3b.

### Bacterial susceptibility to AgNPs

The four Ag colloidal dispersions, as obtained from laser ablation, were used for the antibacterial tests and their MIC and minimum bactericidal concentration (MBC) values were obtained against *E. coli* and *B. subtilis*. To have a known antimicrobial as a reference, we also tested ampicillin against the two bacteria. The results are shown in Table 2.

**Table 2:** MIC and MBC values of the four AgNPs samples against *E. coli* XL1Blue and *B. subtilis* 168 (obtained at least by two independent experiments, each in duplicate).

Antimicrobial	*E. coli*		*B. subtilis*	

	MIC (μg/mL)	MBC (μg/mL)	MIC (μg/mL)	MBC (μg/mL)

AgNPsH_2_Ops	8.4	8.4	1.7	1.7
AgNPsH_2_Ons	9.2	18.4	9.2	18.4
AgNPsLiClps	1.6	2.4	1.0	1.0
AgNPsLiClns	3.7	3.7	1.5	1.5
Ampicillin	14.5	14.5	50.0	75.0

In order to detect the time of appearance of the AgNPs bactericidal effects, *E. coli* cultures with and without AgNPs at the MBC value were prepared in microtiter plates and their optical density and viable count were determined at time zero and after 45 min, 2 h, 3 h and 24 h of incubation at 37 °C. The results of the viable count *E. coli* cultures are reported in Table 3 for ns-ablated AgNPs. *E. coli* cultures tested with AgNPsH_2_Ons (18.4 μg/mL) and AgNPsLiClns (3.7 μg/mL) did not show any increase of optical density at any monitored time, while *E. coli* cultures without additives showed an optical density increase starting from 2 h, until reaching a plateau, as confirmed at 24 h (not shown). According to Table 3, there was a reduction in *E. coli* viability in the presence of AgNPs, starting after 2 h and more evident after 3 h of incubation, until no viable cells were detectable at 24 h.

**Table 3:** Viable count of *E. coli* cultures grown in microtiter plates in the absence and in the presence of AgNPs (MBC value) at different incubation times.

Incubation time (min)	*E. coli* (CFU/mL)	*E. coli* + AgNpsH_2_Ons (CFU/mL)	*E. coli* + AgNpsLiClns (CFU/mL)

0	3.5 × 10^6^	2.8 × 10^6^	3.2 × 10^6^
45	3.3 × 10^6^	2.5 × 10^6^	2.4 × 10^6^
120	5.4 × 10^6^	1.7 × 10^6^	2.0 × 10^6^
180	4.3 × 10^7^	2.3 × 10^5^	1.5 × 10^4^
1440	8.0 × 10^8^	<10^a^	<10^a^

^a^Absence of CFUs growth by plating 0.1 mL of the undiluted culture.

## Discussion

The PLAL preparation method produced AgNPs with a small average diameter (<10 nm, in all cases) and a narrow statistical distribution. Furthermore, their surface cleanliness and the absence of stabilizers or reaction by-products in the colloids allowed the effect of Ag on the bacteria to be isolated and the effect of surface activation by chloride anions on the antimicrobial activity to be studied.

We tested the antimicrobial activity of all the AgNPs preparations presented in Table 1 against two bacteria: *E. coli*, as a reference for Gram-negatives, and *B. subtilis*, as a reference for Gram-positives. We found antibacterial activity against both microorganisms by all four NPs formulations. Regarding their effect on growth inhibition, we obtained MIC values ranging from 1.0 to 8.4 µg/mL with ps-ablated AgNPs. These values were lower for both microorganisms than those obtained with ns-ablated AgNPs (either in water or in LiCl solution). Moreover, AgNPs obtained in the LiCl solution were more effective on both microorganisms than those obtained in pure H_2_O with MIC values in a restricted range of 1.0–3.7 µg/mL (the lowest values obtained for *B. subtilis* at 1.0 and 1.5 µg/mL for ps- and ns-ablated samples, respectively). A similar trend was found with the MBC values, where the lowest values were obtained on both microorganisms with ps-ablated AgNPs in LiCl solution. In general, all of the colloids tested were more effective than the antibiotic tested as a reference (Table 2).

After storing the AgNPs colloidal dispersions for about two months, we repeated the same tests previously performed with fresh samples. The MIC values were confirmed, indicating good stability of the antimicrobial properties.

As previously reported in the literature, AgNPs toxicity depends on various factors, such as size, surface charge, shape, and capping agent [20]. Since PLAL-synthesized NPs are uncapped and spherical, the two last parameters are not considered in the following discussion. Concerning size, it was reported that the activity of AgNPs against both Gram-negative and Gram-positive bacteria increases with decreasing particle diameter [13,28–30]. The bactericidal activity is at least partly related to the direct interaction of the NPs with the cell membrane. In this respect, Morones et al. [13] demonstrated that with Gram-negative bacteria, this type of interaction is size dependent, and it occurs when NPs exhibit a diameter of ≈1–10 nm. In the current study, all our colloids fall in this size range, thus supporting the observed antimicrobial activity.

Beyond size effects, surface charge is considered an important parameter for AgNP activity and colloidal stability. The zeta-potential values of our colloids were always very negative, with the most negative values exhibited by the samples obtained in the presence of LiCl due to the effect of the chloride anions that are strongly adsorbed on the particle surface [35].

In the case of the samples obtained in pure water with ps and ns ablation, no significant difference was found in the NP size. The UV–vis absorption spectra, instead, evidenced a larger content of oxidized silver on the surface of the ps-ablated nanoparticles. This results in the release of more silver ions, which is recognized to be quite important for the antimicrobial activity [15–16]. For the colloids obtained in LiCl solutions obtained from both ps and ns ablation, the higher antimicrobial activity compared with that shown by the corresponding colloid ablated in pure water could be explained on the basis of the increased surface activity. This is evidenced by the increase of both the SERS and catalytic performance of the chloride-activated AgNPs [24]. Actually, the strong adsorption of chloride anions induces the formation of positive surface charges that represent the active sites of the silver surface for the interaction of the nanoparticles with the surrounding environment [35]. These active sites, which really are to be considered positively charged as ascertained for Ag colloids by spectroscopic and theoretical studies [27,35–38], could be efficient for the adsorption of ligand molecules as well as for the action against the microbial cells. Hence, the same mechanism involved in the SERS enhancement of adsorbates and also in the catalytic activity could be responsible for the increased antimicrobial activity observed for the Ag colloids ablated in chloride solution. These positive charges on the surface of AgNPs prepared in LiCl solution could effectively interact with the negatively charged cell surfaces of the examined bacteria.

Since the lack of standardized methods and materials was raised as an issue concerning the evaluation of the antibacterial effect of AgNPs [11], we have compared our data with those obtained in other works, where similar microbial tests on similar organisms were used. Most of the previous studies were performed on AgNPs obtained by chemical methods. Among studies with AgNPs with an average diameter less than 10 nm, the lowest MIC values obtained (to our knowledge) ranged from 6 to 40 µg/mL on *E. coli* strains and from 30 to 40 µg/mL on *B. subtilis* strains [28–30,39]. Only a few studies on the antibacterial properties of PLAL-produced AgNPs are available. Among these, the only data (to the best of our knowledge) on bacterial growth inhibition by the broth dilution method are reported by Pandey et al. [31], who found MIC values of 2 µg/mL against *E. coli* and 5 µg/mL against *B. subtilis* using AgNPs synthesized by PLAL in aqueous medium (10 ns pulse width) with diameters ranging from 9 to 27 nm.

In comparison to the data available on small AgNPs of 3–7 nm, the MIC values of our colloids were comparable [28–29] or lower [30,39] than those previously reported. The MIC values for the AgNPsLiCl sample (1 µg/mL for *B. subtilis*; 1.6 µg/mL for *E. coli*) were the lowest. In particular, for almost all the NP preparations, we found lower MIC values with *B. subtilis* than with *E. coli.* On the other hand, some authors observed a greater antibacterial effect of the AgNPs on Gram-positive than on Gram-negative, or vice versa [40]. Other authors found some strain-specific variation in the sensitivity to AgNPs. Ruparelia et al. [39] reported MIC values ranging from 40 to 180 µg/mL for different strains of *E. coli* and the same MIC value (40 µg/mL) for *B. subtilis* and for the most sensitive *E. coli* strain.

Concerning the AgNPs bactericidal effect, we found that MIC and MBC values were similar (at most doubled) or identical for each NP preparation on each tested microorganism, in agreement with that found on *E. coli* and *B. subtilis* by other authors [30,39].

According to Agnihotri et al. [30], the minimum time necessary to achieve bacteriostatic as well as bactericidal effect (≥99.9% of bacteria are killed) by AgNPs is expected to occur within 3 h. We then tested the time of appearance of the bacteriostatic as well as bactericidal effects of ns-ablated AgNPs at their MBC value on *E. coli* cells. We found that both AgNPs produced in H_2_O and AgNPs produced in LiCl displayed almost similar antibacterial activity: growth inhibition (bacteriostatic effect) occurs immediately after incubation and remains unchanged after 24 h, while the NP killing effect begins after about 2 h and increases at 3 h, with no detectable CFUs after 24 h of incubation (Table 3). Hence, according to our data on ns-ablated AgNPs, the minimum time necessary to achieve bactericidal effect was longer than 3 h. Nevertheless, such AgNPs are very quick at inhibiting bacterial proliferation, when compared to other PLAL-synthesized AgNPs. For example, Pandey et al. [31] observed the growth of different bacterial strains in liquid medium in the presence of PLAL-synthesized ns-ablated AgNPs (diameter 9–27 nm) by measuring the optical density (OD) up to 24 h of incubation. Even if bacterial growth was reduced with respect to the control (without AgNPs), *E. coli* grew in the first five hours, then its growth curve showed an abrupt decrease at nearly 5.5 h, while *B. subtilis* continued to grow until 24 h. Grade et al. [41] monitored the growth of different bacterial strains in liquid medium for 20 h by measuring the OD at 600 nm in the presence of PLAL-prepared ns-ablated AgNPs (diameter 17 nm) at the lowest concentration that inhibits the growth of all the tested bacteria on solid medium (35 µg/mL). They found no reduction of the OD for *E. coli* compared with the control (culture without AgNPs).

## Conclusion

We have synthetized AgNPs with a small average diameter and a narrow size distribution using the pulsed laser ablation in liquid method (PLAL). The diameter of the resulting nanoparticles lies in the size range known to have the highest antimicrobial activity. The AgNPs are effective against both Gram-negative and Gram-positive bacteria, showing MIC values at least comparable or lower than those reported for AgNPs obtained by wet chemical methods as well as by PLAL. An explanation is proposed for the higher antimicrobial activity exhibited by Ag colloids ablated in chloride solution in comparison with colloids ablated in pure water. This explanation is based on the increased surface reactivity of the metal surface due to the presence of positively charged active sites. The higher antimicrobial activity shown by the ps-ablated colloids with respect to the ns-ablated colloids could, instead, be justified by the larger amount of oxidized silver present on the particle surface.

## Experimental

### Preparation of AgNPs

The ablation step was performed with two different lasers: a mode-locked Nd:YAG laser emitting ps pulses at 1064 nm (EKSPLA PL2143A, repetition rate 10 Hz, pulse width 25 ps) and a Q-switched Nd:YAG laser emitting ns pulses at 1064 nm (Quanta System CLS 400, repetition rate 10 Hz, pulse width 25 ns). The pulse energy and spot size at the target were fixed at 15 mJ and 1.4 mm and 100 mJ and 1.6 mm, for ps and ns ablation, respectively. The ablation was performed either in doubly deionized water (18.2 MΩ·cm at 25 °C) or in 1 mM water solutions of LiCl, in a 1 × 1 cm quartz cell and the liquid column above the target was 2 cm. Taking water absorption at 1064 nm into account, the laser fluence at the target corresponds to 0.7 J/cm^2^ and 4 J/cm^2^ for ps and ns PLAL, respectively. The Ag target was purchased from Goodfellow and the LiCl from Sigma-Aldrich (purity 99%). Prior to ablation, all the materials required for the process were sterilized in an autoclave.

The TEM samples were obtained by dropping a small amount of colloid onto carbon-coated copper grids and allowing it to evaporate. The images were recorded with a Philips CM12 at 120 kV. The nanoparticle mean diameter was determined by fitting the measured statistical distributions (obtained with more than 2000 counts) with a lognormal function having σ_−_ and σ_+_ 1/*e* left and right half widths, respectively.

ICP-AES measurements of the Ag concentration in the colloids were performed with a Varian 720-ES inductively coupled plasma atomic emission spectrometer.

The zeta potential of the colloids in water and in chloride solution was measured with a ZetasizerNano ZS90 (Malvern Instruments). The errors in the zeta-potential values are the standard deviation of the mean value, as obtained by the software supplied with the Malvern instrument.

### Bacterial strains and growth conditions

The *Escherichia coli* strain XL1Blue (Stratagene, La Jolla, CA, USA) and the *Bacillus subtilis* strain 168 [42] were used to test the antimicrobial activity of AgNP preparations as representatives of Gram-negative and Gram-positive bacteria, respectively.

The cells of both bacteria were grown aerobically at 37 °C: *E. coli* in liquid or solid Luria broth (LB) complex medium [43], *B. subtilis* 168 in Nutrient Broth (NB, OXOID) and Nutrient Agar (NA, OXOID).

### Measurement of antimicrobial activity

*E. coli* XL1Blue and *B. subtilis* 168 were used to test the bacterial inhibitory activity of the four AgNP preparations by determining their MIC and MBC. As an antimicrobial reference, ampicillin was used for both *E. coli* and *B. subtilis*. To determine the MIC, the broth microdilution method in standard microtiter plates was used as previously described [44]. In this case, bacterial cells were grown in LB or NB and collected at the exponential growth phase to be diluted in LB 2× or NB 2× until the OD at 590 nm was 0.05, corresponding to a cell density of approximately (2–4) × 10^6^ colony forming units (CFUs) per mL. The antimicrobials were serially diluted in sterile water (AgNPs-H_2_O, ampicillin) or in sterile 1 mM aqueous LiCl solutions (AgNPs-LiCl) and then added to an equal volume of bacterial suspension in LB 2× or NB 2× for a final volume of 250 µL in each microtiter well to obtain the desired final concentration. Initial tenfold dilutions were used to identify the range of concentrations including the MIC for each antimicrobial; then, starting from this range, 1:2, 1:2.5 and 1:3 serial dilutions were used to identify the MIC value with higher accuracy. The viable count of the initial bacterial inoculum was determined by serial dilutions and plating. Microtiter plates were incubated at 37 °C, with shaking at 100 rpm; the plates were read at 590 nm in a microtiter plate reader (Immunella S, GDV, Rome, Italy) at time zero and after 24 h of incubation. The MIC was considered as the lowest concentration of an antimicrobial agent that completely inhibited growth by optical density measurement (no absorbance increase was observed in the microtiter well after 24 h of incubation). For each test, at least two independent experiments were performed in duplicate. Growth control, consisting of a bacterial inoculum in LB or NB medium, both in water and in 1 mM LiCl solution with no test compounds, and sterility controls, consisting of growth medium and medium plus AgNPs at the tested concentrations, were always included in each test.

To determine the MBC, after 24 h of incubation for the MIC determination, the cultures in microtiter wells with an antimicrobial concentration higher or equal to the MIC value were serially diluted, plated on solid LB or NA without antimicrobials, and incubated 24 h at 37 °C. Then, the survivors were counted as CFU/mL. The MBC was identified as the lowest concentration of the antibacterial agent that reduced the viability of the initial bacterial inoculum by ≥99.9%.

## References

[1] McFarland A D, Van Duyne R P (2003). Nano Lett.

[2] Wang M, De Vivo B, Lu W, Muniz-Miranda M (2014). Appl Spectrosc.

[3] Zoppi A, Trigari S, Giorgetti E, Muniz-Miranda M, Alloisio M, Demartini A, Dellepiane G, Thea S, Dobrikov G, Timtcheva I (2013). J Colloid Interface Sci.

[4] Arvizo R R, Bhattacharyya S, Kudgus R A, Giri K, Bhattacharya R, Mukherjee P (2012). Chem Soc Rev.

[5] Pergolese B, Muniz-Miranda M, Bigotto A (2007). Chem Phys Lett.

[6] Li C, Yamauchi Y (2013). Phys Chem Chem Phys.

[7] Ataee-Esfahani H, Imura M, Yamauchi Y (2013). Angew Chem, Int Ed.

[8] Wang L, Yamauchi Y (2013). J Am Chem Soc.

[9] Muniz-Miranda M, Gellini C, Salvi P R, Pagliai M (2009). J Raman Spectrosc.

[10] Caporali S, Moggi-Cecchi V, Muniz-Miranda M, Pagliai M, Pratesi G, Schettino V (2012). Meteorit Planet Sci.

[11] Rizzello L, Pompa P P (2014). Chem Soc Rev.

[12] Bellissima F, Bonini M, Giorgi R, Baglioni P, Barresi G, Mastromei G, Perito B (2014). Environ Sci Pollut Res.

[13] Morones J R, Elechiguerra J L, Camacho A, Holt K, Kouri J B, Tapia Ramírez J, Yacaman M J (2005). Nanotechnology.

[14] Kim J S, Kuk E, Yu K N, Kim J-H, Park S J, Lee H J, Kim S H, Park Y K, Park Y H, Hwang C-Y (2007). Nanomedicine: NBM.

[15] Xiu Z-m, Zhang Q-b, Puppala H L, Colvin V L, Alvarez P J J (2012). Nano Lett.

[16] Ivask A, El Badawy A, Kaweeteerawat C, Boren D, Fischer H, Ji Z, Chang C H, Liu R, Tolaymat T, Telesca D (2014). ACS Nano.

[17] Feng Q L, Wu J, Chen G Q, Cui F Z, Kim T N, Kim J O (2000). J Biomed Mater Res.

[18] Lok C-N, Ho C-M, Chen R, He Q-Y, Yu W-Y, Sun H, Tam P K-H, Chiu J-F, Che C-M (2006). J Proteome Res.

[19] Rai M, Yadav A, Gade A (2009). Biotechnol Adv.

[20] Marambio-Jones C, Hoek E M V (2010). J Nanopart Res.

[21] Silver S (2003). FEMS Microbiol Rev.

[22] Sharma V K, Yngard R A, Lin Y (2009). Adv Colloid Interface Sci.

[23] Sintubin L, Verstraete W, Boon N (2012). Biotechnol Bioeng.

[24] Muniz-Miranda M (2013). J Raman Spectrosc.

[25] Nikolov A S, Nedyalkov N N, Nikov R G, Atanasov P A, Alexandrov M T, Karashanova D B (2012). Appl Phys A.

[26] Zeng H, Du X-W, Singh S C, Kulinich S A, Yang S, He J, Cai W (2012). Adv Funct Mater.

[27] Giorgetti E, Marsili P, Muniz-Miranda M, Gellini C, Giammanco F (2014). Appl Phys A.

[28] Lu Z, Rong K, Li J, Yang H, Chen R (2013). J Mater Sci: Mater Med.

[29] Martínez-Castañòn G A, Niño-Martínez N, Martínez-Gutierrez F, Martínez-Mendoza J R, Ruiz F (2008). J Nanopart Res.

[30] Agnihotri S, Mukherji S, Mukherji S (2014). RSC Adv.

[31] Pandey J K, Swarnkar R K, Soumya K K, Dwivedi P, Kumar Singh M, Sundaram S, Gopal R (2014). Appl Biochem Biotechnol.

[32] Giorgetti E, Marsili P, Giammanco F, Trigari S, Gellini C, Muniz-Miranda M (2015). J Raman Spectrosc.

[33] Mao X L, Ciocan A C, Borisov O V, Russo R E (1998). Appl Surf Sci.

[34] Giorgetti E, Giammanco F, Marsili P, Giusti A (2011). J Phys Chem C.

[35] Muniz-Miranda M, Sbrana G (1996). J Raman Spectrosc.

[36] Muniz-Miranda M, Pagliai M (2013). J Phys Chem C.

[37] Pagliai M, Muniz-Miranda F, Schettino V, Muniz-Miranda M, Starov V, Griffiths P (2012). Competitive Solvation and Chemisorption in Silver Colloidal Suspensions. Progress in Colloid and Polymer Science.

[38] Muniz-Miranda M, Pagliai M, Muniz-Miranda F, Schettino V (2011). Chem Commun.

[39] Ruparelia J P, Chatterjee A K, Duttagupta S P, Mukherji S (2008). Acta Biomater.

[40] Wrótniak-Drzewiecka W, Gaikwad S, Laskowski D, Dahm H, Niedojadlo J, Gade A, Rai M (2014). Austin J Biotechnol Bioeng.

[41] Grade S, Eberhard J, Wagener P, Winkel A, Sajti C L, Barcikowski S, Stiesch M (2012). Adv Eng Mater.

[42] Anagnostopoulos C, Spizizen J (1961). J Bacteriol.

[43] Miller J H (1972). Experiments in molecular genetics.

[44] Colzi I, Troyan A N, Perito B, Casalone E, Romoli R, Pieraccini G, Škalko-Basnet N, Adessi A, Rossi F, Gonnelli C (2015). Eur J Pharm Biopharm.

